# The Response of *Paraburkholderia terrae* Strains to Two Soil Fungi and the Potential Role of Oxalate

**DOI:** 10.3389/fmicb.2018.00989

**Published:** 2018-05-29

**Authors:** Irshad Ul Haq, Reto Daniel Zwahlen, Pu Yang, Jan Dirk van Elsas

**Affiliations:** ^1^Microbial Ecology, Groningen Institute for Evolutionary Life Sciences, University of Groningen, Groningen, Netherlands; ^2^Department of Microbiology, University of Haripur, Haripur, Pakistan; ^3^Molecular Microbiology, Groningen Biomolecular Sciences and Biotechnology Institute, University of Groningen, Groningen, Netherlands

**Keywords:** oxalic acid, chemotaxis, fungal exudates, *Paraburkholderia terrae*, signaling

## Abstract

Fungal-associated *Paraburkholderia terrae* strains in soil have been extensively studied, but their sensing strategies to locate fungi in soil have remained largely elusive. In this study, we investigated the behavior of five mycosphere-isolated *P. terrae* strains [including the type-3 secretion system negative mutant BS001-Δ*sctD* and the type strain DSM 17804^T^] with respect to their fungal-sensing strategies. The putative role of oxalic acid as a signaling molecule in the chemotaxis toward soil fungi, as well as a potential carbon source, was assessed. First, all *P. terrae* strains, including the type strain, were found to sense, and show a chemotactic response toward, the different levels of oxalic acid (0.1, 0.5, and 0.8%) applied at a distance. The chemotactic responses were faster and stronger at lower concentrations (0.1%) than at higher ones. We then tested the chemotactic responses of all strains toward exudates of the soil fungi *Lyophyllum* sp. strain Karsten and *Trichoderma asperellum* 302 used in different dilutions (undiluted, 1:10, 1:100 diluted) versus the control. All *P. terrae* strains showed significant directed chemotactic behavior toward the exudate source, with full-strength exudates inciting the strongest responses. In a separate experiment, strain BS001 was shown to be able to grow on oxalate-amended (0.1 and 0.5%) mineral medium M9. Chemical analyses of the fungal secretomes using proton nuclear magnetic resonance (^1^H NMR), next to high-performance liquid chromatography (HPLC), indeed revealed the presence of oxalic acid (next to glycerol, acetic acid, formic acid, and fumaric acid) in the supernatants of both fungi. In addition, citric acid was found in the *Lyophyllum* sp. strain Karsten exudates. Given the fact that, next to oxalic acid, the other compounds can also serve as C and energy sources for *P. terrae*, the two fungi clearly offer ecological benefits to this bacterium. The oxalic acid released by the two fungi may have primarily acted as a signaling molecule, and, as a “second option,” a carbon source for *P. terrae* strains like BS001.

## Introduction

Bacteria in soil, most often, encounter conditions of nutrient (carbon source) scarcity (Van Elsas et al., 2007). Hence, the hospitable microhabitats at plants and fungi, where carbonaceous compounds become available, constitute attractive refuges. To localize such potential niches, soil bacteria depend on the genetic traits that enable them to perceive potential signals, leading to chemotaxis and guiding them toward hospitable microenvironments. Bacteria that have a symbiotic phase included in their lifestyle are particularly dependent on processes of recognition and positive movement. In previous studies, several fungi in soil have been found to release carbon- and energy-yielding compounds like oxalic acid (Sullivan et al., 2012). Such compounds are used by soil fungi to scavenge metals and release inorganic nutrients (Van Hees et al., 2006; Adeleke et al., 2012). In some plant-associated fungi as well as in fungi involved in the decay of litter and wood, oxalic acid can even form complexes – in the form of oxalate bound to cations like Ca^2+^ – on the hyphal tips (Dutton and Evans, 1996; Bhatti et al., 1998; Harrold and Tabatabai, 2006; Van Hees et al., 2006; Heller and Witt-Geiges, 2013). A limited range of soil bacteria is known to be able to use oxalic acid and its complexes as a potential carbon source, in sites under the direct influence of fungi (Bravo et al., 2013) or plants (Franceschi and Nakata, 2005). Interestingly, oxalic acid has recently been shown to act as a signaling molecule for bacteria of the genus *Collimonas* (Rudnick et al., 2015). In addition, it was shown to serve as a carbon source for several *Paraburkholderia* species, including the plant-associated *Paraburkholderia phytofirmans* PsJN (Kost et al., 2014).

The genus *Paraburkholderia* encompasses different species, of which some can interact with soil fungi. Examples are *Paraburkholderia terrae*, in particular strain BS001 and the related strains BS110, BS007, and BS437 (Nazir et al., 2012, 2014). These strains have been shown to harbor genetic systems that furnish the capacity to obtain ecological benefits from fungi in soil (Nazir et al., 2012; Haq et al., 2014). Among such systems, those responsible for chemotaxis and flagellar movement are of prime importance (Yang et al., 2017), as they underlie the active exploration by the bacterium, via fungal-assisted migration, of the soil for hospitable space, in which sensing the putative chemoattractants released by soil fungi can play a role. Boersma et al. (2010) had already shown that *Lyophyllum* sp. strain Karsten could release glycerol into the medium it is grown in. Later, Nazir et al. (2013) provided evidence for the contention that the interaction between *Lyophyllum* sp. strain Karsten and *P. terrae* BS001 (in liquid microcosms) promoted the release of glycerol by the fungus, spurring the growth of the bacterium. Strain BS001 was also able to grow actively in minimal medium supplemented with glycerol as the sole carbon source (Nazir et al., 2013). However, evidence for the production of oxalic acid by *Lyophyllum* sp. strain Karsten (as well as *Trichoderma asperellum* 302), and its putative role in their interactions with *P. terrae* strains BS001, BS110, BS007, and BS437, has been lacking.

In this study, we tested the hypothesis that *Lyophyllum* sp. strain Karsten and *T. asperellum* 302 exude different small carbonaceous compounds, including oxalic acid, which attract, and offer ecological benefits to, the fungal-interactive *P. terrae* strains BS001, BS110, BS007, and BS437, next to the type strain DSM 17804^T^. We used simple microcosm setups to test bacterial behavior, and performed proton nuclear magnetic resonance (^1^H NMR) and high-performance liquid chromatography (HPLC) to investigate the nature of the fungal-released chemical compounds. We further hypothesized that oxalic acid acts primarily as a signaling molecule for the fungal-associated *B terrae* strains.

## Materials and Methods

### Bacterial Strains, Growth Conditions, and Media

In this study, we used four fungal-interactive *Paraburkholderia* strains, i.e., BS001, BS110, BS007, and BS437, next to a type-3 secretion system (TTSS) (Δ*sctD*) mutant of strain BS001 (Yang et al., 2016) and type strain DSM 17804^T^. All strains were maintained at -80°C, and then kept as inoculum sources on R2A agar at 25°C. All *P. terrae* cultures used were grown overnight in Luria–Bertani (LB) broth at 28°C, with shaking at 180 rpm. At harvest, the cultures were subjected to centrifugation at 12,000 ×*g* for 3 min. The bacterial pellets were then double-washed with sterile saline (0.85% NaCl solution). For all experiments on chemotaxis, cultures were centrifuged at 1,057 ×*g* for 20 min and the resulting pellets washed twice with morpholine ethane sulfonic (MES) acid buffer, containing 1 g of KH_2_PO_4_ and 1 g (NH_4_)_2_SO_4_ per L; pH 5.6 (Rudnick et al., 2015).

### Chemotaxis Assays

The chemotaxis assays were performed in M9-G agar Petri plates, with the final concentration of the agar set at 0.25% [M9 agar for testing of swimming motility (M9A-S)]. M9-G medium consisted of 6.76 g of Na_2_HPO_4_, 3 g of KH_2_PO_4_, 0.5 g NaCl, and 1 g of NH_4_Cl per L MilliQ water (pH 6.8), supplemented with 0.5% glycerol and 2 mL (per L) of filter-sterilized (0.2 μm) 1 M MgSO_4_ and 100 μL (per L) of 1 M CaCl_2_.

To set up the experiments with oxalic acid as well as fungal exudates, molten M9-G agar [1.2% (w/v)] was mixed 1:1 with either oxalic acid [final levels 0.1–0.5 and 0.8% (w/v)] or different levels of fungal exudates (undiluted, 1:10, and 1:100 diluted). Controls contained either no oxalic acid (oxalic acid control) or no exudates (exudate control). Following solidification of the agar, rectangular (15 mm × 5 mm) stripes were severed from the media and introduced as “target stripes” in the chemotaxis assay Petri plates.

The washed bacterial cells resuspended in MES buffer were adjusted to establish approximate cell numbers of 10^7^ CFU/mL. The suspensions were then introduced as 5-μL stripes onto the Petri plates at distances of about 15 mm from, and parallel with, the agar stripes containing either oxalic acid [0 (controls), 0.1, 0.5, and 0.8% (w/v)] or fungal exudates [zero (controls), undiluted, 1:10, and 1:100 diluted]. The Petri plates were incubated at 25°C for up to 48 h, and readings [distances traveled in millimeters, in the forward (“toward”) and backward (“away from”) directions] were taken manually at set times.

### Growth of *P. terrae* Strain BS001 on Oxalate as the Sole Carbon and Energy Source

Washed cells of *P. terrae* strain BS001 were introduced, at estimated levels of 10^7^ cells/mL, into triplicate (per treatment) 100-mL Erlenmeyer flasks containing 20 mL of M9 medium supplemented with zero (negative control), 0.1, or 0.5% of oxalate or glucose (positive control). The flasks were incubated at 28°C, with shaking at 150 rpm, and samples were aseptically taken at set times for CFU enumerations. The samples were dilution-plated on LB agar plates and CFU counts determined following incubation of the plates at 28°C for up to 1 week.

### Fungal Strains, Growth Media, and Conditions to Obtain Exudates

For long-term maintenance, *Lyophyllum* sp. strain Karsten and *T. asperellum* 302 were maintained in autoclaved distilled water. For routine use, the fungi were refreshed once a month on oat flake agar (OFA) plates. For analysis of their respective secretomes, both fungal strains were first grown for 48–72 h in oat flake medium at 28°C with shaking. The well-grown fungal cultures were then centrifuged at 2,655 ×*g* (FA-45-30-11 rotor, Eppendorf) for 10 min and the pellets washed twice with M9. M9 medium supplemented with 1% [w/v] sodium propionate (pH 4.8) was then used for fungal growth for the preparation of fungal secretomes. Thus, Erlenmeyer flasks containing 50 mL of medium were inoculated with the respective strains and incubated for two weeks at 28°C, with shaking.

### Harvesting Fungal Exudates and Sample Preparation for ^1^H NMR

Well-grown 50-mL cultures of both *Lyophyllum* sp. strain Karsten and *T. asperellum* 302 were harvested by centrifugation at 4,226 × g (F-35-6-30 rotor, Eppendorf) for 10 min. The resulting supernatants were then filtered through 0.2 μm filters (Whatman; FP 30/0.2 CA-S), to remove fungal hyphae and debris. The filtrates containing the exudates were stored at -20°C. For ^1^H NMR analyses, samples were subsequently defrosted at room temperature. To 570 μL of filtrate, 30 μL of D_2_O containing 3-(trimethylsilyl)-1-propanesulfonic acid sodium salt (DSS; 0.1 mM final concentration) were added (as internal standard). To minimize pH-based peak shifts, the pH for all samples was adjusted to 7.0 with either HCl or NaOH. For ^1^H NMR data acquisition, aliquots of 600 μL were transferred to Bruker NMR tubes.

### ^1^H NMR Data Acquisition and Analysis

The ^1^H NMR spectra were acquired on a Bruker DRX400 spectrometer 500-MHz (Bruker Spectrospin, Toronto, ON, Canada). The ^1^H NMR spectra were further processed and analyzed using MestReNova (version 9.0.0-12821) software. For identification, spectra obtained were compared with spectra of known compounds using the Human Metabolome Database (HMDB).

### High-Performance Liquid Chromatography (HPLC)-Based Analysis of Fungal Exudates

Oxalic acid levels were determined in the exudates of both fungi, using a C18 reverse phase column (id 5 μm; d 4.6 mm × 250 mm) on a Shimadzu UPLC device (Shimadzu Corp., Kyoto, Japan). An isocratic method was chosen, using a 25 mM K-phosphate buffer with a pH of 2.4 as the mobile phase. The pump flow was set at 1.5 mL/min and the column temperature at 30°C. Then, samples at 4°C (20 μL) were run [sample injection followed 30 s after run initiation with a total length of 14 min]. The chromatogram was subsequently evaluated at 210 nm. Peak areas were extracted using the Shimadzu software. The oxalic acid concentration was derived using an external standard at concentrations of 0.01, 0.05, 0.10, 0.25, 0.5, and 1.0% (w/v). Corresponding peaks were further identified using 0.1% oxalic acid internal sample spiking.

### Analysis of Putative Oxalate Degradation Pathways in *Paraburkholderia* Genomes

We used the MetaCyc database and specifically examined the available *P. terrae* genomes (strains BS001, BS110, BS007, and BS437) for oxalate degradation pathways. The *P. terrae* BS001 genome was published in Haq et al. (2014), and the genomes of strains BS007, BS110, and BS437 in Pratama et al. (2017). We did a directed search in the aforementioned genomes hosted at MicroScope (Vallenet et al., 2013) for the presence and absence of any of the five known pathways (I–V) of oxalate degradation.

### Statistical Analyses

All chemotaxis experiments had four biological replications. Full factorial ANOVA was performed using RStudio Version 0.99.893 – © 2009–2016 RStudio, Inc. The input data consisted of the distances (measured in mm) traveled by the bacterial strains in two directions (“toward” and “away”). The confidence interval of 95% was used in all the analyses.

## Results

### Chemotactic Behavior of Fungal-Interactive *Paraburkholderia terrae* Strains Exposed to Various Concentrations of Oxalic Acid

**FIGURE 1:**
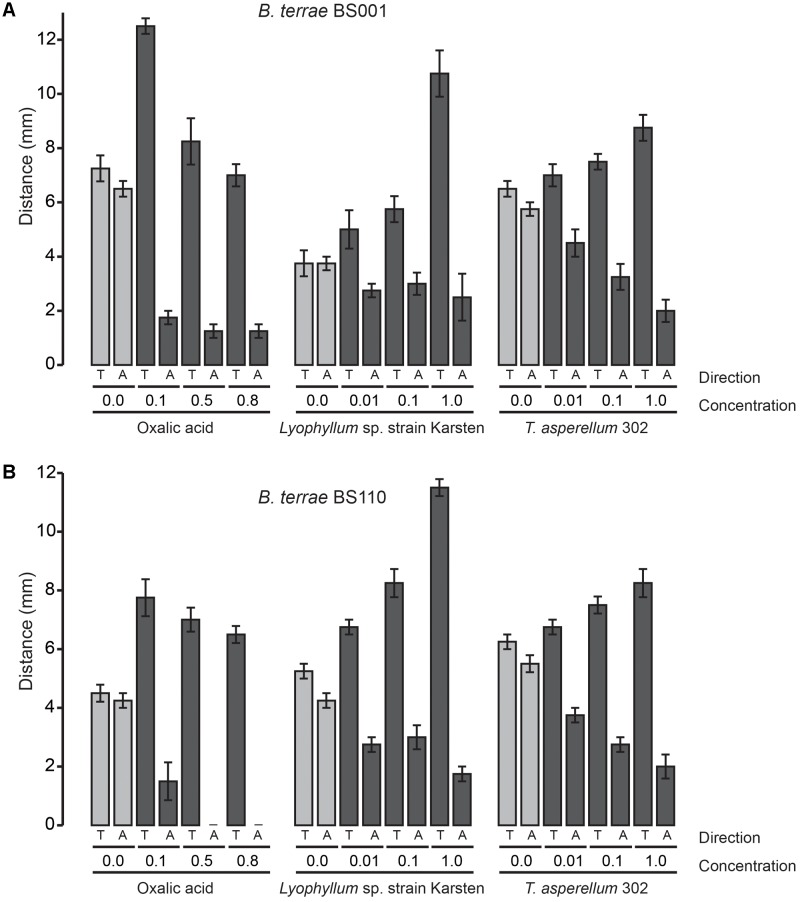
Chemotaxis (swimming) of *P. terrae* strains BS001 and BS110 toward oxalate and exudates of *Lyophyllum* sp. strain Karsten and *T. asperellum* 302 on 0.25% (w/v) M9 medium. **(A)** The response of *P. terrae* BS001 to either oxalate or exudates of both fungal strains. **(B)** The response of *P. terrae* BS110 either oxalate or exudates of both fungal strains. The *Y*-axis represents the distance in mm, whereas the *X*-axis shows the direction of taxis (“toward” is denoted by “T” and “away” by “A”) and concentration of oxalate or levels of fungal exudates. Data are representative of four biological replicates.

**FIGURE 2:**
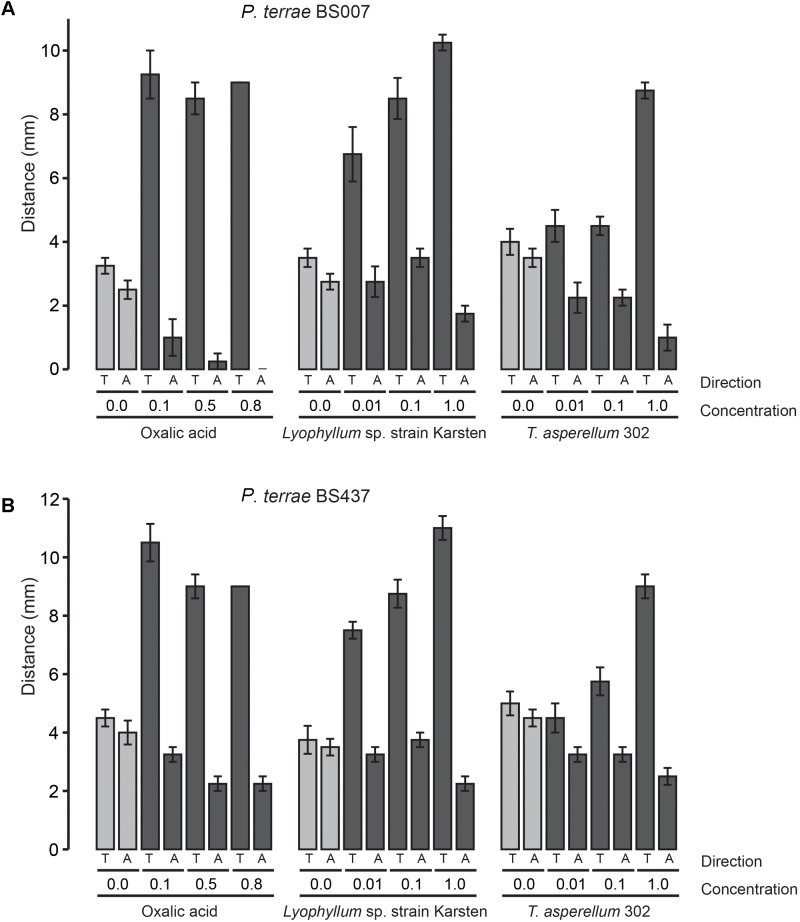
Chemotactic (swimming) behavior of *P. terrae* strains BS007 and BS437 toward oxalate and exudates of *Lyophyllum* sp. strain Karsten and *T. asperellum* 302 on 0.25% (w/v) M9 medium. **(A)** The response of *P. terrae* BS007 to either oxalate or exudates of both fungal strains. **(B)** The response of *P. terrae* BS437 to either oxalate or exudates of both fungal strains. The *Y*-axis represents distance in mm, whereas the *X*-axis shows the direction of taxis (toward denoted by “T” and away denoted by “A”) and concentration of oxalate or levels of exudates. Data are representative of four biological replicates.

**FIGURE 3:**
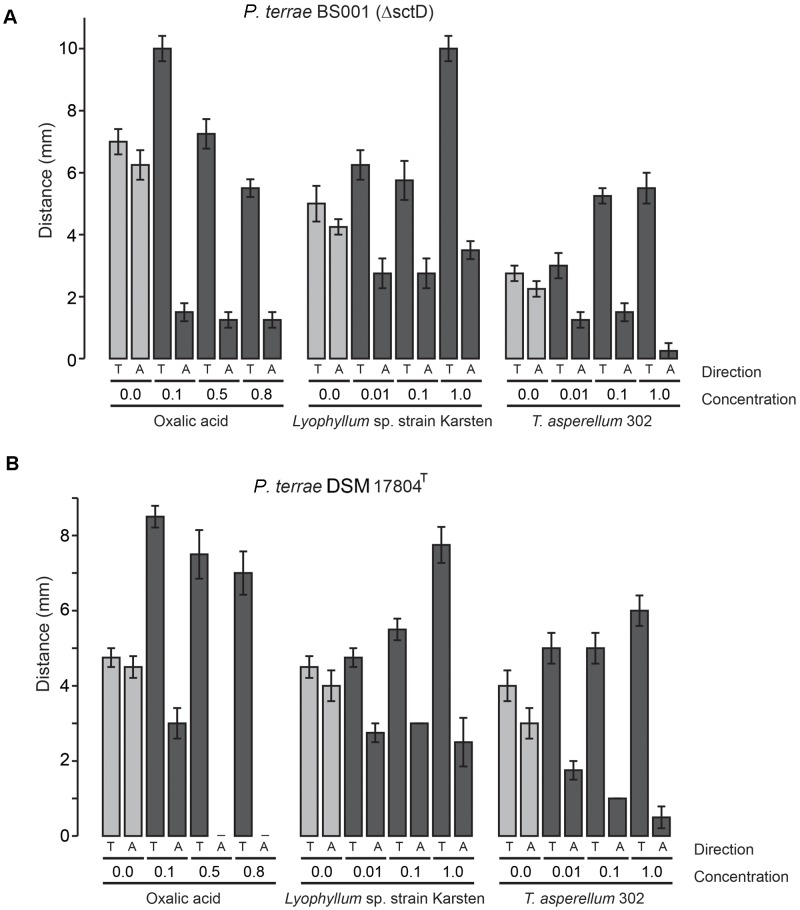
Chemotactic (swimming) behavior of *P. terrae* strains BS001-Δ*sctD* and DSM 17804^T^ toward oxalate and exudates of *Lyophyllum* sp. strain Karsten and *T. asperellum* 302 on 0.25% (w/v) M9 medium. **(A)** The response of *P. terrae* BS001-Δ*sctD* to either oxalate or exudates of both fungal strains. **(B)** The response of *P. terrae* DSM 17804^T^ to either oxalate or exudates of both fungal strains The *Y*-axis represents the distance in mm, whereas the *X*-axis shows the direction of taxis (toward denoted by “T” and away denoted by “A”) and concentration of oxalate or levels of exudates. Data are representative of four biological replicates.

To understand the ecological behavior at fungi of the selected *P. terrae* strains, we tested their chemotactic (swimming) responses (**Figures 1**–**3** and **Supplementary Figure S1**) on M9-G agar. Oxalic acid in different concentrations [0.1, 0.5, 0.8, and 0% (control)] was used as the putative chemoattractant in the used agar stripes. Full factorial ANOVA (**Supplementary Table S1**) of the collective data revealed that the factors oxalic acid level and movement type (toward or away from the attractant) were the key drivers of the chemotactic responses of all *P. terrae* strains. This included the type strain, which *a priori* had not been selected for fungal interactivity. First, without any added oxalate, the swimming behavior of all strains was “random” in the sense that the movement “toward” versus “away” was never different (*P* > 0.05). Second, the chemotactic responses of all strains toward the oxalic acid source were significantly faster and higher (*P* < 0.05) than away from it, clearly suggesting that positive chemotaxis took place. Remarkably, the responses were stronger at the lowest concentration of oxalic acid (0.1%) than at the two higher concentrations (*P* < 0.05).

### Chemotactic Responses of Fungal-Interactive *Paraburkholderia terrae* Strains Toward Exudates of *Lyophyllum* sp. Strain Karsten and *T. asperellum* 302

We then tested the chemotactic responses of all *P. terrae* strains upon confrontation with exudates from *Lyophyllum* sp. strain Karsten as well as *T. asperellum* 302, at the different levels. First, the swimming behavior of all strains in response to control stripes (no fungal exudate added) was undirected, as all strains apparently explored the milieu around the inoculum similarly in both directions (toward versus away; *P* > 0.05). Interestingly, all *P. terrae* strains showed a strong chemotactic response toward the M9 agar stripes containing fungal exudates (full-strength, 1:10 diluted and 1:100 diluted), as compared to the movement away (*P* < 0.05). A full factorial ANOVA (**Supplementary Table S2**) of the data showed that the movement type (“toward” versus “away”), fungal type, bacterial strain and exudate level in the stripes were the factors driving the chemotactic behavior of all *P. terrae* strains (including the type strain DSM 17804^T^). The chemotactic responses were stronger toward the exudates of *Lyophyllum* sp. strain Karsten than to those of *T. asperellum* 302 (data of all strains are shown in **Figures 1**–**3**). There were differences in the movement of the strains toward the exudates (full-strength) of both fungi, with strains BS110, BS437, BS001, and BS007 being more “proactive” than strain DSM 17804^T^.

With respect to the responses to *T. asperellum* 302, the different *P. terrae* strains reacted differently to the same concentrations of the exudates, with strains BS110, BS437, BS001, and BS007 being more “avid” responders than strains DSM 17804^T^ and BS001-Δ*sctD*. ANOVA (**Supplementary Table S2**) of the data revealed that three factors, i.e., exudate level, taxis direction (toward/away) and *P. terrae* strain, were the key variables that drove the chemotactic responses. Thus, there were significant differences (*P* < 0.05) in the responses of all *P. terrae* strains toward full-strength exudates (1%) compared to the diluted ones (0.01 and 0.1%) and the control (0%). At higher concentration (1%), the bacterial taxis was significantly one-directional (toward the stripes) and stronger (*P* < 0.05).

### Identification and Quantification of Oxalic Acid in the Exudates of *Lyophyllum* sp. Strain Karsten and *T. asperellum* 302

Previous work (Boersma et al., 2010) already analyzed *Lyophyllum* sp. strain Karsten exudates by ^1^H NMR; however, this method cannot detect oxalic acid. Hence, here we examined the exudates of *Lyophyllum* sp. strain Karsten as well as *T. asperellum* 302 for the presence of oxalic acid using HPLC analyses over a C18 reverse phase column (id 5 μm; d 4.6 mm × 250 mm). Control runs of progressively increasing levels of pure oxalic acid revealed the appearance of progressively bigger peaks, at retention time 2.4 min, in the resulting chromatograms (**Supplementary Figure S2**). The exudates from both fungi were then examined. Both showed the appearance of peaks at the exact same retention time, and thus oxalic acid was presumably produced. Quantification of the peaks revealed that considerable amounts had been released by both fungi into the propionate-amended M9 medium, with final oxalic acid levels being in the range 0.075–0.093% (w/v; **Table 1**). To validate the identification of the respective peaks in the chromatogram, we spiked the respective fungal exudates with 0.1% (w/v) of oxalic acid in the same HPLC run (**Supplementary Figure S2**). Indeed, the added 0.1% oxalic acid about doubled the peak weight and so the measured amount of oxalic acid. Together, our results suggest that oxalic acid is indeed released by both fungal types, in rather similar quantities, under the applied conditions.

**Table 1:** Quantitative analysis of the exudates of *Lyophyllum* sp. strain Karsten and *Trichoderma asperellum* 302 for the identification of oxalic acid, using high-performance liquid chromatography (HPLC). Oxalic acid was used as an external standard and M9 medium as a negative control.

Compounds	Retention time (min)	Area	Height	Concentration % (w/v)
MilliQ	0.00	0	0	0.000
Oxalic acid_0.01%	2.48	386,101	27,450	0.010
Oxalic acid_0.01%	2.48	369,726	27,375	0.010
Oxalic acid_0.05%	2.39	5,071,290	562,392	0.051
Oxalic acid_0.05%	2.39	5,033,482	567,899	0.051
Oxalic acid_0.10%	2.39	9,400,274	1,084,105	0.099
Oxalic acid_0.10%	2.38	9,960,133	1,074,788	0.102
M9 medium	2.37	86,273	12,120	0.001
M9 medium	2.37	103,940	14,645	0.001
M9+0.1% Oxalic acid	2.37	8,007,944	1,290,413	0.092
M9+0.1% Oxalic acid	2.38	8,026,472	1,301,304	0.092
*Lyophyllum* sp. strain Karsten	2.41	7,795,023	719,831	0.090
*Lyophyllum* sp. strain Karsten	2.41	6,417,830	710,125	0.074
*Lyophyllum* sp. strain Karsten 1:5	2.36	3,554,342	518,881	0.041
*Lyophyllum* sp. strain Karsten 1:5	2.36	3,243,405	494,120	0.037
*Lyophyllum* sp. strain Karsten 1:10	2.36	2,205,528	388,395	0.025
*Lyophyllum* sp. strain Karsten 1:10	2.35	2,217,147	359,645	0.025
*T. asperellum* 302	2.40	8,137,144	683,484	0.093
*T. asperellum* 302	2.40	7,592,703	680,421	0.087
*T. asperellum* 302 1:5	2.36	1,576,896	286,151	0.018
*T. asperellum* 302 1:5	2.35	1,500,720	262,248	0.017
*T. asperellum* 302 1:10	2.36	847,009	162,324	0.010
*T. asperellum* 302 1:10	2.35	848,337	162,203	0.010


### ^1^H NMR-Based Analyses of the Exudates Released by *Lyophyllum* sp. Strain Karsten in Propionate-Amended M9 Medium

**FIGURE 4:**
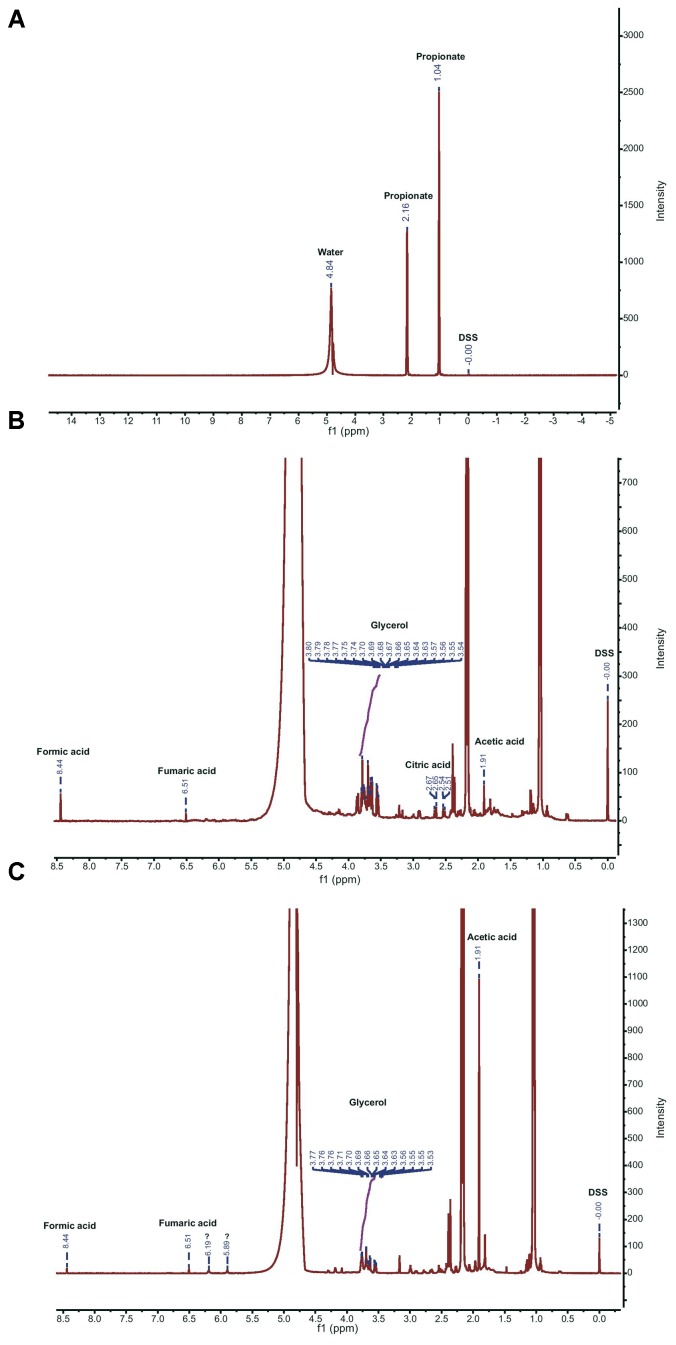
Proton NMR spectra of M9 medium, and fungal exudates. **(A)** M9 spectrum showing peaks corresponding to water and propionate. **(B)** Full spectrum of exudates of *Lyophyllum* sp. strain Karsten. **(C)** Full spectrum of exudates of *T. asperellum* 302. 3-(trimethylsilyl)-1-propanesulfonic acid sodium salt (DSS) was used as the internal standard (0.0 ppm). Corresponding peaks of compounds in exudates of *Lyophyllum* sp. strain Karsten **(B)** and *T. asperellum* 302 **(C)** are annotated with their names. Unknown peaks are denoted by (?).

To underpin the chemotaxis findings, we then re-analyzed the exudates of *Lyophyllum* sp. strain Karsten, using ^1^H NMR. The resulting spectra showed the existence of various peaks, indicating that *Lyophyllum* sp. strain Karsten had released various small compounds. The control M9+propionate medium, as well as that containing potential fungal exudates, both showed two large peaks at 1.04 and 2.16 ppm, respectively, which were both attributed to propionate (**Figure 4A**). The potential exudate spectra further revealed a multiplet corresponding to glycerol, as found earlier (Boersma et al., 2010; from 3.54 to 3.80 ppm). Expectedly, acetic acid (1.91 ppm) and formic acid (8.44 ppm) were also detected (**Figure 4B**). Moreover, another multiplet (2.51; 2.54 and 2.65/2.67 ppm) was identified as citric acid/citrate. Finally, a peak probably representing fumaric acid (6.51 ppm) stood out. Both citric and fumaric acid had not been detected previously (Boersma et al., 2010). Several other small peaks could not be attributed to any known compound or metabolite. Thus, *Lyophyllum* sp. strain Karsten releases a suite of compounds/metabolites, including glycerol, acetate, formate, citrate, and fumarate, into its milieu that might be perceived and used by the fungal-interactive bacteria *P. terrae* BS001, BS110, BS007, and BS437 as potential carbon and energy sources.

### ^1^H NMR-Based Analysis of the Exudates Released by *Trichoderma asperellum* 302 Into Propionate-Amended M9 Medium

We then analyzed the exudates of *T. asperellum* 302, using ^1^H NMR, in order to find any commonalities and/or differences with the exudates of *Lyophyllum* sp. strain Karsten. First, glycerol was also found to be released by *T. asperellum* 302, as evidenced by the finding of a multiplet in the range reported in the foregoing (**Figure 4C**). Then, we also found peaks representative of acetic acid (1.91 ppm) and formic acid (8.44 ppm). Interestingly, fumaric acid/fumarate (6.51 ppm) was, presumably, also detected. Next to these broad commonalities in the metabolites released by both fungi, there were two unknown and as-yet-unidentified peaks (at 5.89 and 6.19 ppm), which were absent from the spectra obtained with *Lyophyllum* sp. strain Karsten. On the other hand, we did not find the multiplet representing citric acid/citrate. Thus, the ecological opportunities offered by *T. asperellum* 302 to its associated *P. terrae* strains were, to some extent, similar to those of *Lyophyllum* sp. strain Karsten, with some differences as suggested by the presence of unidentified peaks.

### Growth of *Paraburkholderia terrae* BS001 on Oxalate-Supplemented M9 Medium

**FIGURE 5:**
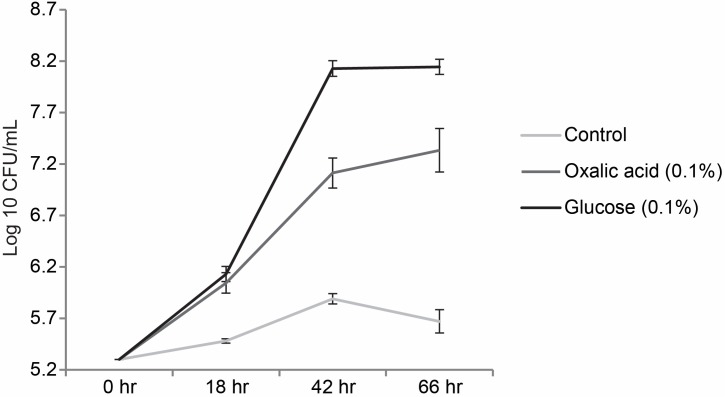
Growth curve of *P. terrae* BS001 grown on M9 medium supplemented with oxalate; represents the growth patterns of *P. terrae* BS001 on M9 medium supplemented with 0.1% of glucose, 0.1% oxalate [w/v] and no carbon source. The *Y*-axis represents CFU/mL on a log 10 scale, while the *X*-axis shows the times in hours. Data are representative of three biological replicates.

We then tested whether or not oxalic acid could serve as the sole carbon and energy source for the growth of *P. terrae* BS001 in M9 medium. Unsupplemented M9 medium was used as the negative and M9 medium supplemented with (0.5%) glucose as the positive control. The negative control did not support growth of strain BS001, as the CFU counts – from the initial 5.30 log 10 CFU/mL – increased only slightly over the 66 h incubation time, indicating residual growth (data not shown). *P. terrae* BS001 grown in M9+0.5% glucose showed a significantly increased (*P* < 0.05) final population density of 7.89 log 10 CFU/mL after 66 h of incubation. On 0.5% oxalate, we observed a progressive increase in the initial cell density, from about 5.30 log 10 to 6.20 log 10 CFU/mL (after 18 h) and then to 7.68 log 10 CFU/mL (42 h), after which it slightly dropped back to 6.86 log 10 CFU/mL at 66 h of incubation (data not shown). On M9 medium supplemented with 0.1% oxalate (w/v), the CFU counts of strain BS001, from an initial 5.30 log 10 CFU/mL, also increased significantly (*P* < 0.05), reaching 7.33 log 10 CFU/mL after 66 h of incubation (**Figure 5**). The overall results suggest that *P. terrae* BS001 can use oxalic acid as a carbon and energy source when supplied as a single carbonaceous compound in M9 medium.

### Genetic Potential for Oxalate Utilization in *P. terrae* Strains

We analyzed the genomes of *P. terrae* BS001, BS110, BS437, and BS007 to find genes that are potentially involved in the utilization and uptake of oxalic acid or its complexes (pathways I–V). Although we did not find any genes (as per the annotation) that are predicted to encode enzymes involved in oxalate degradation pathway I, we found genes encoding enzymes involved in pathways II and III in all *P. terrae* strains (**Table 2**). Moreover, we did find a cluster of five genes, which was recently reported to be upregulated in the transcriptome of strain BS001 during its interaction (physical contact stage) with *Lyophyllum* sp. strain Karsten (Haq et al., 2017), across all *P. terrae* strains (recently described by Pratama et al., 2017). The cluster contains a gene encoding a so-called “cupin” protein that was postulated to be acting as oxalate oxidase and/or oxalate carboxylase and may potentially be involved in the degradation of oxalate to formate and H_2_O_2_ as per the pathways IV and V.

**Table 2:** The list of oxalate degrading pathways according to MetaCyc. The potential presence (+) or absence (-) of oxalate degradation pathways is shown in the respective *Paraburkholderia terrae* strains. Cupin domain containing proteins are postulated to be acting either as oxalate oxidase or oxalate decarboxylase.

Strains	Pathway I^(a)^	Pathway II^(b)^	Pathway III^(c)^	Pathway IV^(d)^	Pathway V^(e)^
*Paraburkholderia terrae* BS001	-	+	+	±	±
*Paraburkholderia terrae* BS110	-	+	+	±	±
*Paraburkholderia terrae* BS007	-	+	+	±	±
*Paraburkholderia terrae* BS437	-	+	+	±	±
*Paraburkholderia terrae* DSM 17804^T^	N/A	N/A	N/A	N/A	N/A

*N/A, not applicable, as genome information not available at the time of writing. “+” indicates presence, “-” indicates absence, and “±” presence or absence (cupin domain containing proteins; described down the line). ^*(a)*^Single reaction pathway that involves the direction conversion of oxalate to two CO2 molecules that is catalyzed by oxalate oxidoreductase in the presence of two oxidized ferredoxin [iron–sulfur] cluster. ^*(b)*^Consists of three reactions resulting in the conversion of oxalate to formate, involving oxalate CoA–transferase, oxalyl–CoA decarboxylase, and formyl–CoA transferase. ^*(c)*^The third pathway concerning oxalate degradation has two possible outcomes, where oxalyl–CoA is either converted to formate and subsequently to NADH or to glyoxalate that goes to glycolate and glyoxalate degradation pathway. ^*(d)*^The fourth pathway involves only one reaction where oxalate is converted to hydrogen peroxide via oxalate oxidase. ^*(e)*^The fifth pathway involves only one reaction where oxalate is converted to formate via oxalate decarboxylase. Cupin: either acting as oxalate oxidase (monocupin) or oxalate decarboxylase (bicupin; Tanner et al., 2001). Based on the presence of cupin in the genomes of P. terrae strains, they are proposed to have roles in oxalate degradation*.

## Discussion

The ecological benefits offered by soil fungi to bacteria living in their vicinity have been addressed previously (De Boer et al., 2005; Warmink and van Elsas, 2009; Nazir et al., 2010; Pion et al., 2013; Stopnisek et al., 2016). A key model “system,” featuring *P. terrae* BS001 and a range of soil fungi (Nazir et al., 2014), including *Lyophyllum* sp. strain Karsten, has previously been interrogated with respect to the potential mechanisms involved in the bacterial–fungal interaction (Warmink and van Elsas, 2008, 2009; Nazir et al., 2013; Haq et al., 2014). However, questions on the fungal responsiveness of other *P. terrae* strains, such as BS110, BS007, and BS437, have remained unanswered. Moreover, any information regarding the type strain DSM 17804^T^ has been lacking so far. Hence, there is a perceived lack of information with respect to the commonality of fungotropic chemotactic behavior within the species *P. terrae*. In this study, we thus examined the chemotactic behavior of a range (BS110, BS007, BS437, BS001, BS001-Δ*sctD* and DSM 17804^T^) of *P. terrae* strains toward exudates collected from the basidiomycete *Lyophyllum* sp. strain Karsten and the ascomycete *T. asperellum* 302, as well as oxalate. In recent findings, by Yang et al. (2016), the TTSS was found to have a helper effect in the migration of *P. terrae* toward fungal mycelia in soil microcosms, and therefore the inclusion of the TTSS-impaired mutant in the current study was deemed necessary to ascertain its potential role in chemotaxis. Concerning the signaling mechanism that might be employed, there are examples of a wide number of methyl-accepting chemotaxis proteins (MCPs) in bacteria such as VfcB and VfcB2 of *Vibrio fischeri*, that act as fatty acid chemoreceptors (Nikolakakis et al., 2016).

First, the analyses of the fungal exudates using proton ^1^H NMR and HPLC revealed the two fungi to release remarkably similar amounts of a few detectable small carbonaceous compounds, including the major compounds oxalate, glycerol, acetate, and formate, and presumably fumarate. The finding of oxalate was revealing and added this compound to the list of potential chemoattractants, and possibly carbon sources, for *P. terrae* (further discussed below). On the other hand, we found the differential presence of citrate (at *Lyophyllum* sp. strain Karsten) and an unidentified compound (at *T. asperellum* 302). Overall, one may conclude that a suite of compounds, next to glycerol, are released by both fungi, potentially serving as chemoattractants for *P. terrae*.

Remarkably, all *P. terrae* strains, including the type strain DSM 17804^T^, indeed were found to move, in a concerted fashion, toward the exudates of both fungi, with lower responses at the higher dilutions, and an overall difference between the two fungi. A slight decrease of migrational competence was found in the TTSS mutant strain, the exact cause of which remained unexplored. Further, the fact that the migrational behavior of the type strain was akin that of the other *P. terrae* strains indicated that – within the limitations of this study – *P. terrae*, in contrast to what was previously thought, might constitute a “tight” group of fungal-interactive organisms. This is opposed to the hypothesis that strain BS001 and its relatives are outliers within this species (compared to the type strain), as – in contrast to the type strain – they were isolated on the basis of their fungal interactivity (Warmink and van Elsas, 2008; Nazir et al., 2013, 2014).

Furthermore, given the fact that the two fungi indeed may release several of the aforementioned carbonaceous compounds into the soil, fungal-responsive *Paraburkholderia* species (broadly speaking, the species *P. terrae*) have likely adapted to migrate toward the fungal mycelia, thus reaping the ecological benefits of the utilization of the different compounds (e.g., glycerol, acetate, formate, fumarate, and oxalate; see below), for growth and survival.

Concerning the role of oxalic acid as a determinant of the behavior of the tested *P. terrae* strains, the data clearly indicated that all *P. terrae* strains responded to the oxalic acid/oxalate stemming from the agar stripes in the chemotaxis setup, even in the presence of glycerol in the M9 agar medium. Thus, even with a highly utilizable source of carbon and energy, the *P. terrae* cells migrated toward the source of oxalate. A question with respect to oxalate availability arises here, as (dissociated) oxalate was the most prominent chemical form under our experimental conditions (pH 6.8). The finding that the oxalate showed the highest attractant activity when at the lower “stripe” concentration (0.1%) was difficult to explain. However, the chemical interplay with (bivalent) cations present in the agar (affecting oxalate diffusion), in particular Ca^2+^ (0.1 mM), is complex and difficult to model. Remarkably, in a recent study with *Collimonas fungivorans* Ter331, an increasing concentration of oxalate in the medium was found to prompt a “lethargic” chemotactic response by bacteria, compared to a more active one at lower concentration (Rudnick et al., 2015). Notwithstanding its clear effect as a chemoattractant for all *P. terrae* strains when present as the sole carbonaceous compound, we did not address here to what extent the fungal-released oxalate was a major or minor chemoattractant in the presence of the other fungal-released compounds (such as glycerol). Clearly, this poses a complex question, which was not answered here.

The potential oxalotrophy of *P. terrae* was then investigated on the basis of growth experiments with *P. terrae* BS001. Indeed, we found that *P. terrae* BS001 was able to grow on M9 medium supplemented with defined concentrations of oxalic acid. This stands in contrast to previous findings (using Biolog assays) where *P. terrae* strains were reportedly unable to utilize oxalate (Nazir et al., 2012). However, Kost et al. (2014) reported that a range of plant-beneficial *Burkholderia* species (now split into two genera, *Burkholderia* and *Paraburkholderia*), including close relatives of *P. terrae*, namely *P. phytofirmans*, *P. xenovorans*, *P. hospita*, and *P. caribensis*, were able to grow on minimal medium containing oxalic acid as the sole carbon source. In contrast, the plant-pathogenic *Burkholderia glumae*, *B. gladioli*, and *B. plantarii* and human-pathogenic strains of the *B. cepacia* complex were unable to use oxalate as the carbon source. The here collected data (**Table 2**) suggest that *P. terrae* should be added to the list of oxalotrophic organisms. What are the ecological consequences of this finding? In the light of the rather poor energetic value of oxalate (Schneider et al., 2012), it may serve as a last-resort carbon source in the presence of other such sources. However, oxalotrophy should not be excluded as a true option for energy generation under conditions of starvation. In previous work, fungal-released oxalate in soil has been hypothesized to act as a virulence factor (thus promoting cellular activity) and as a helper in lignin degradation (Dutton and Evans, 1996; Heller and Witt-Geiges, 2013). Also, plant-released oxalate may act in calcium homeostasis, in repellance of herbivores or in toxic metal (e.g., aluminum) chelation (Franceschi and Nakata, 2005; Klug and Horst, 2010). Here, we posit that, apart from such anthropogenically inspired function attributions, fungal-released oxalate acts as a key “last-resort” carbon and energy source, selecting bacterial species such as *P. terrae* in the mycosphere upon the arisal of strongly carbon-limited conditions.

Supportive of the tenet of active oxalate oxidation was the fact that, indeed, in all *P. terrae* strains, systems for the catabolism (and uptake) of oxalate were found. The presence of genes encoding key enzymes of degradation pathways II and III, and that of the “cupin” (oxalate oxidase/carboxylase) thus suggested all strains have the genetic information enabling them to utilize oxalate. Moreover, we recently observed that the gene encoding the cupin protein was upregulated by *P. terrae* under the influence of growing mycelia of *Lyophyllum* sp. strain Karsten under soil-mimicking conditions (Haq et al., 2017). However, with respect to the latter phenomenon, further experiments would be needed to have deeper insight into the specific role of the cupin protein.

On the basis of the data presented herein, we further hypothesize that the oxalate/oxalic acid exuded by fungi in soil may be used by locally occurring *P. terrae* cells as a signaling molecule which enables to locate a colonizable surface, next to a plethora of nutrients. Concerning the latter, Boersma et al. (2010) previously quantified the glycerol levels in the exudates of *Lyophyllum* sp. strain Karsten in propionate-amended M9 medium, and found levels of approximately 2 mM. In that study, glycerol was presumed to be the major bacterium-feeding substrate coming from the fungus. Here, the estimated oxalic acid levels were approximately 7.5–7.9 mM, or three- to fourfold more. However, the fact that glycerol holds more energy than oxalate, and can easily diffuse (passively) into bacterial cells, suggests that it serves as a more accessible, and hence preferable, carbon and energy source than oxalate. Similarly, all other compounds released by the two fungi may also serve as “better” nutrients. In this theory, one may envision diauxic trophic behavior of *P. terrae* strains like BS001 in the mycosphere, with oxalate indeed acting as a last-resort nutrient. Furthermore, in the behavioral response to the two fungi, a chemoattractant/signaling function of oxalic acid cannot also be excluded.

## Conclusion

Our results confirm that the nutritional conditions offered by two diverse soil fungi had key commonalities. In the light of the finding of oxalate, glycerol, and three organic acids (next to one or more unknown compounds) in the fungal secretomes, these conditions are of a complex nature. Yet, they offer the baseline against which interactions of the two fungi with *P. terrae* strains take place. We argue that our findings are indicative of a highly complex interplay, which, to expand it to natural soil environment, needs further investigation. Possibly, several diauxic shifts, as incited by presumably different and varying levels of the fungal-released compounds, occur over time in the fungal-interactive strains, and so it will be a challenge for future studies to precisely determine the sequence of events that take place in the chemotactic behavior of *P. terrae* at the two fungi.

## Author Contributions

IH conceived the idea, performed the experiments, analyzed the data, and wrote the manuscript. RZ performed the HPLC and subsequent analyses. PY provided the mutant strain. JE conceived the idea and wrote the manuscript.

## Conflict of Interest Statement

The authors declare that the research was conducted in the absence of any commercial or financial relationships that could be construed as a potential conflict of interest.

## Supplementary Material

FIGURE S1 A representative figure of the chemotactic response of *P. terrae* toward oxalate.

FIGURE S2 High-performance liquid chromatography (HPLC) chromatograms representing oxalic acid peaks of: oxalic acid [0.1 and 0.25% (w/v)] as a positive control, M9 medium as a negative control, and M9 medium spiked with oxalic acid [0.1% (w/v)], *Lyophyllum* sp. strain Karsten exudates and *Lyophyllum* sp. strain Karsten exudates spiked with oxalic acid (0.1% [w/v]; “sample A” means *Lyophyllum* sp. strain Karsten exudates sample “A,” *Trichoderma asperellum* 302 exudates and *T. asperellum* 302 exudates spiked with oxalic acid [0.1% (w/v)] “Tri B” means *T. asperellum* 302 exudates “B.” At the end, the calibration curve is provided.

TABLE S1 Full factorial ANOVA testing for the effects of bacterial strains, oxalic acid concentration, movement direction, and their interaction on the distance traveled in the chemotaxis experiment on M9-G medium. Statistical significance codes: 0 “^∗∗∗^” 0.001 “^∗∗^” 0.01 “^∗^” 0.05 “.” 0.1 “ ” 1.

TABLE S2 Full factorial ANOVA testing for the effects of bacterial strains, fungal strains, exudates level (conc.), movement direction, and their interaction on the distance traveled in the chemotaxis experiment on M9-G medium. Statistical significance codes: 0 “^∗∗∗^” 0.001 “^∗∗^” 0.01 “^∗^” 0.05 “.” 0.1 “ ” 1.
