# Supplementary figures and images for: The Response of Paraburkholderia terrae Strains to Two Soil Fungi and the Potential Role of Oxalate
Source: Front Microbiol. 2018 May 29;9:989. doi: 10.3389/fmicb.2018.00989 (PMC5986945; doi:10.3389/fmicb.2018.00989)

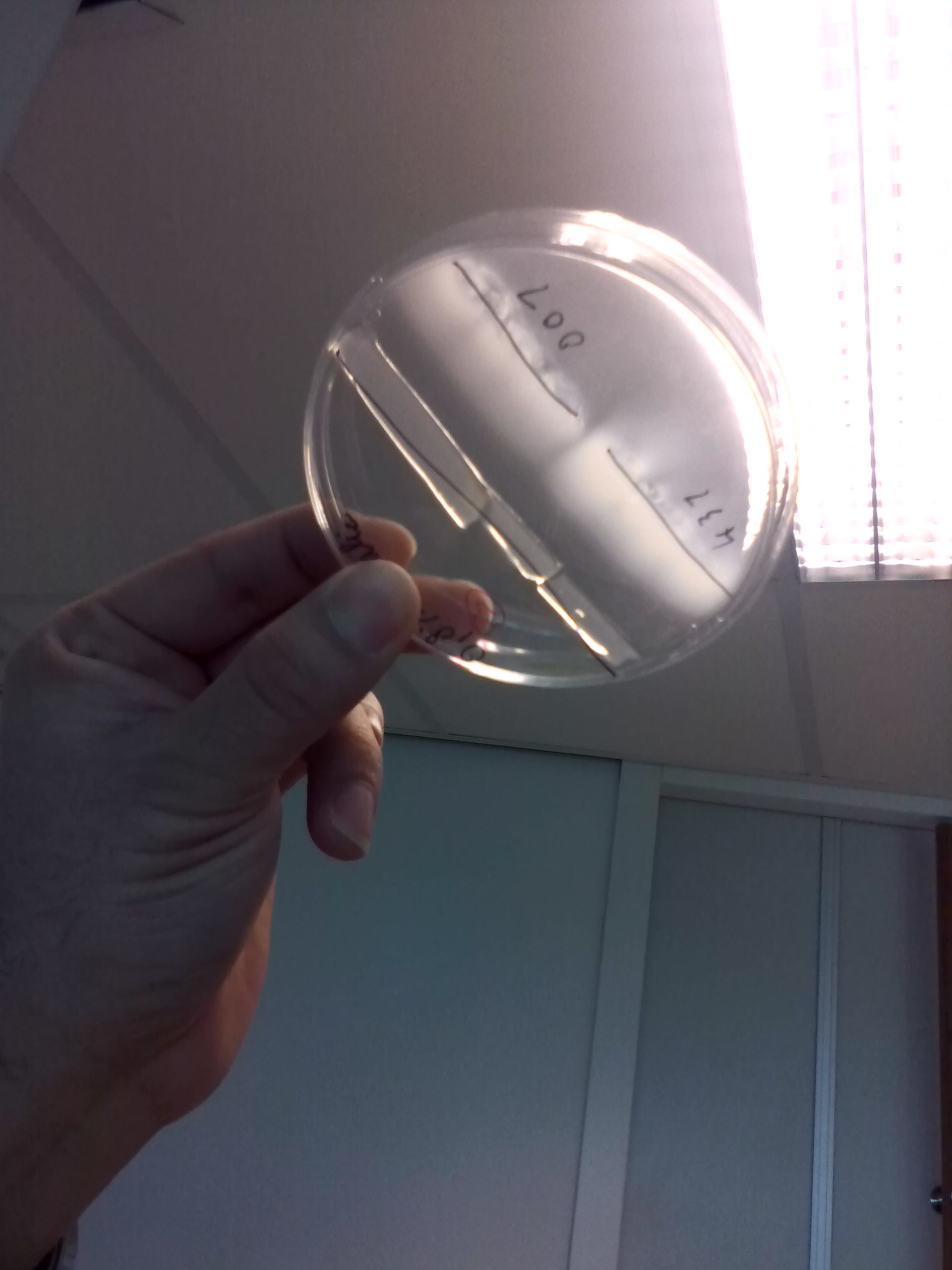

Supplement: FIGURE S1 — A representative figure of the chemotactic response of P. terrae toward oxalate. [file Image_1.JPEG]
