# Supplementary material for: The Response of Paraburkholderia terrae Strains to Two Soil Fungi and the Potential Role of Oxalate
Source: Front Microbiol. 2018 May 29;9:989. doi: 10.3389/fmicb.2018.00989 (PMC5986945; doi:10.3389/fmicb.2018.00989)
Supplement: FIGURE S2 — High-performance liquid chromatography (HPLC) chromatograms representing oxalic acid peaks of: oxalic acid [0.1 and 0.25% (w/v)] as a positive control, M9 medium as a negative control, and M9 medium spiked with oxalic acid [0.1% (w/v)], Lyophyllum sp. strain Karsten exudates and Lyophyllum sp. strain Karsten exudates spiked with oxalic acid (0.1% [w/v]; “sample A” means Lyophyllum sp. strain Karsten exudates sample “A,” Trichoderma asperellum 302 exudates and T. asperellum 302 exudates spiked with oxalic acid [0.1% (w/v)] “Tri B” means T. asperellum 302 exudates “B.” At the end, the calibration curve is provided. [file Image_2.PDF]

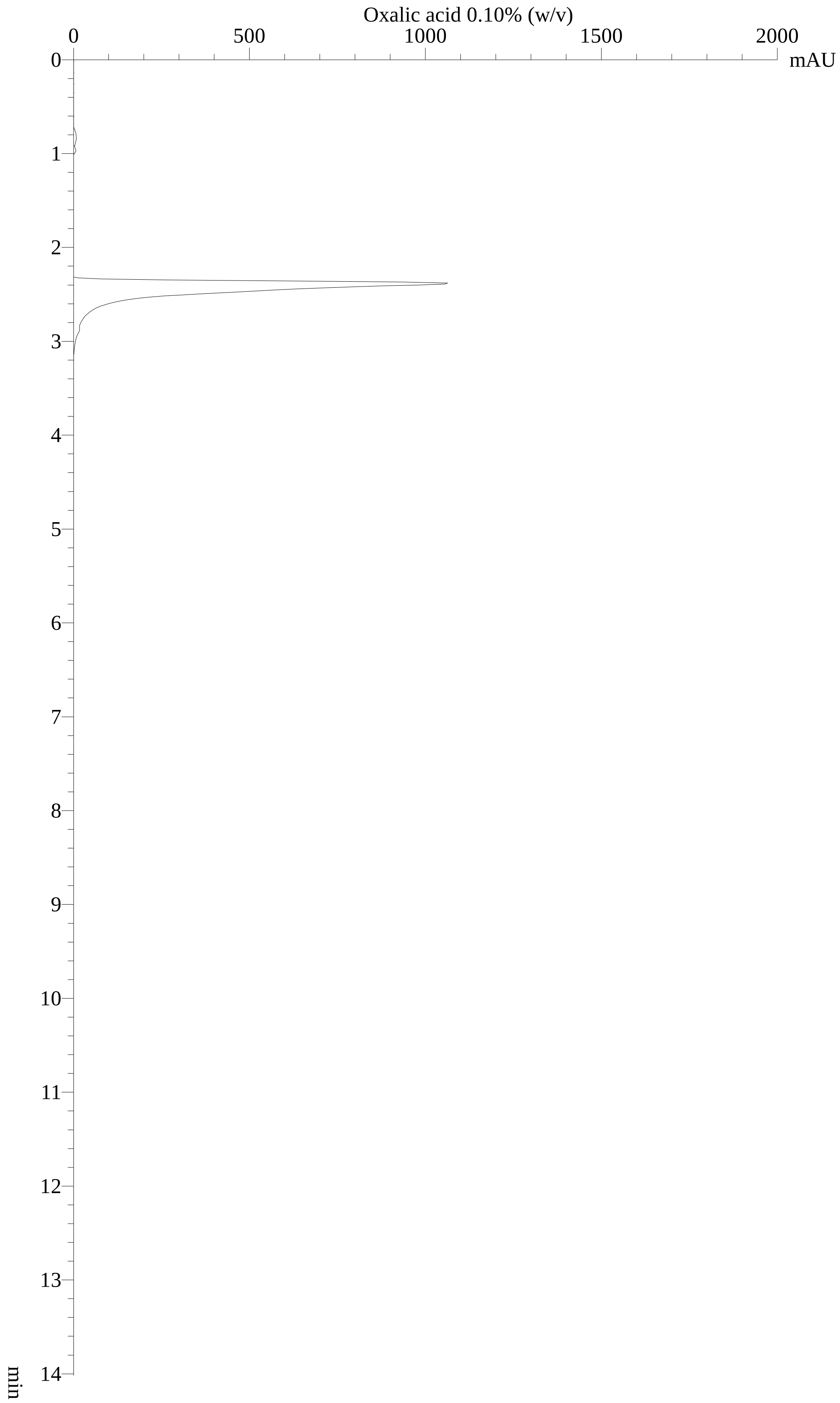
Oxalic acid 0.10% (w/v)
0
500
1000
1500
2000
mAU
0
1
2
3
4
5
6
7
8
9
10
11
12
13
14
min

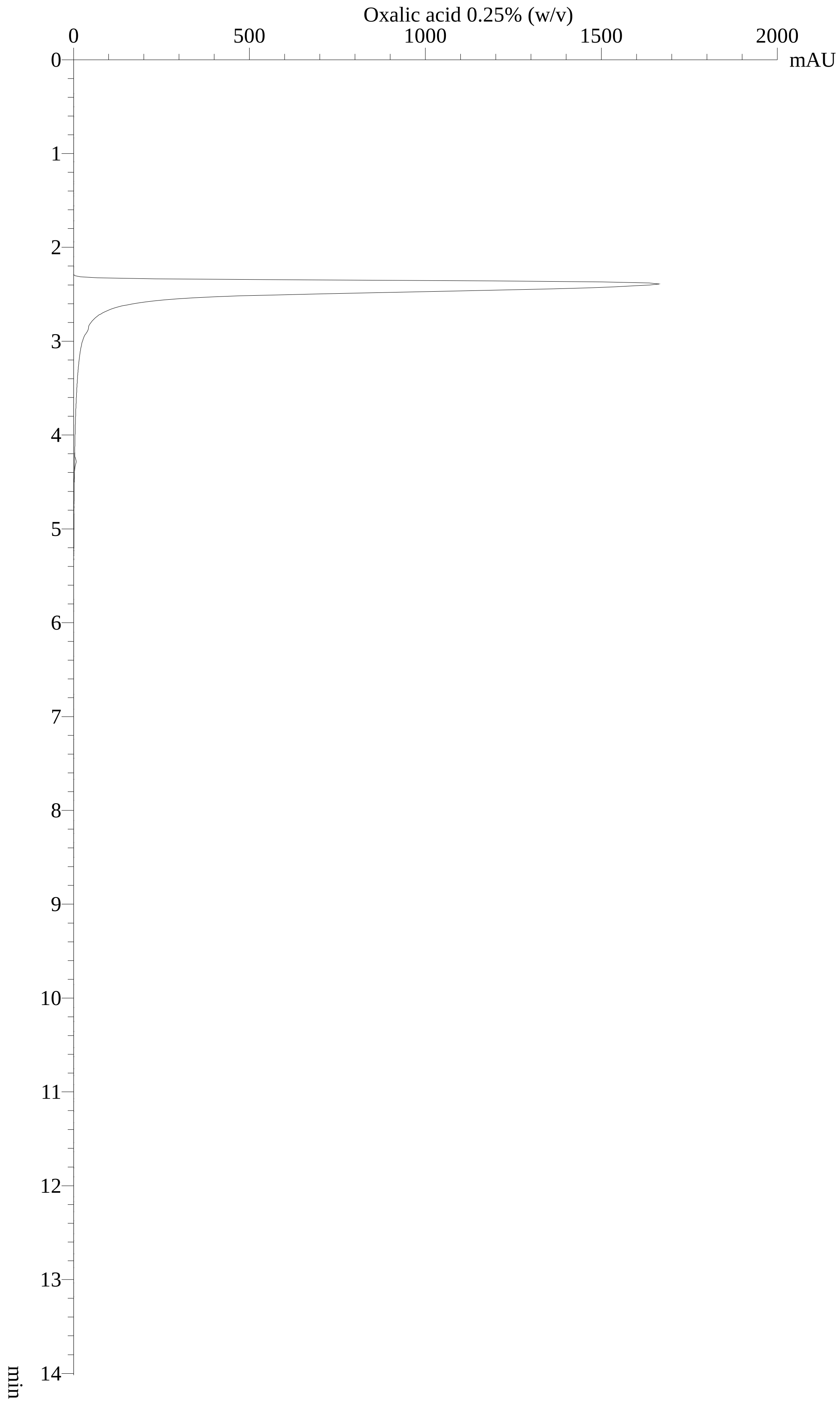
Oxalic acid 0.25% (w/v)
0
500
1000
1500
2000
mAU
0
1
2
3
4
5
6
7
8
9
10
11
12
13
14
min

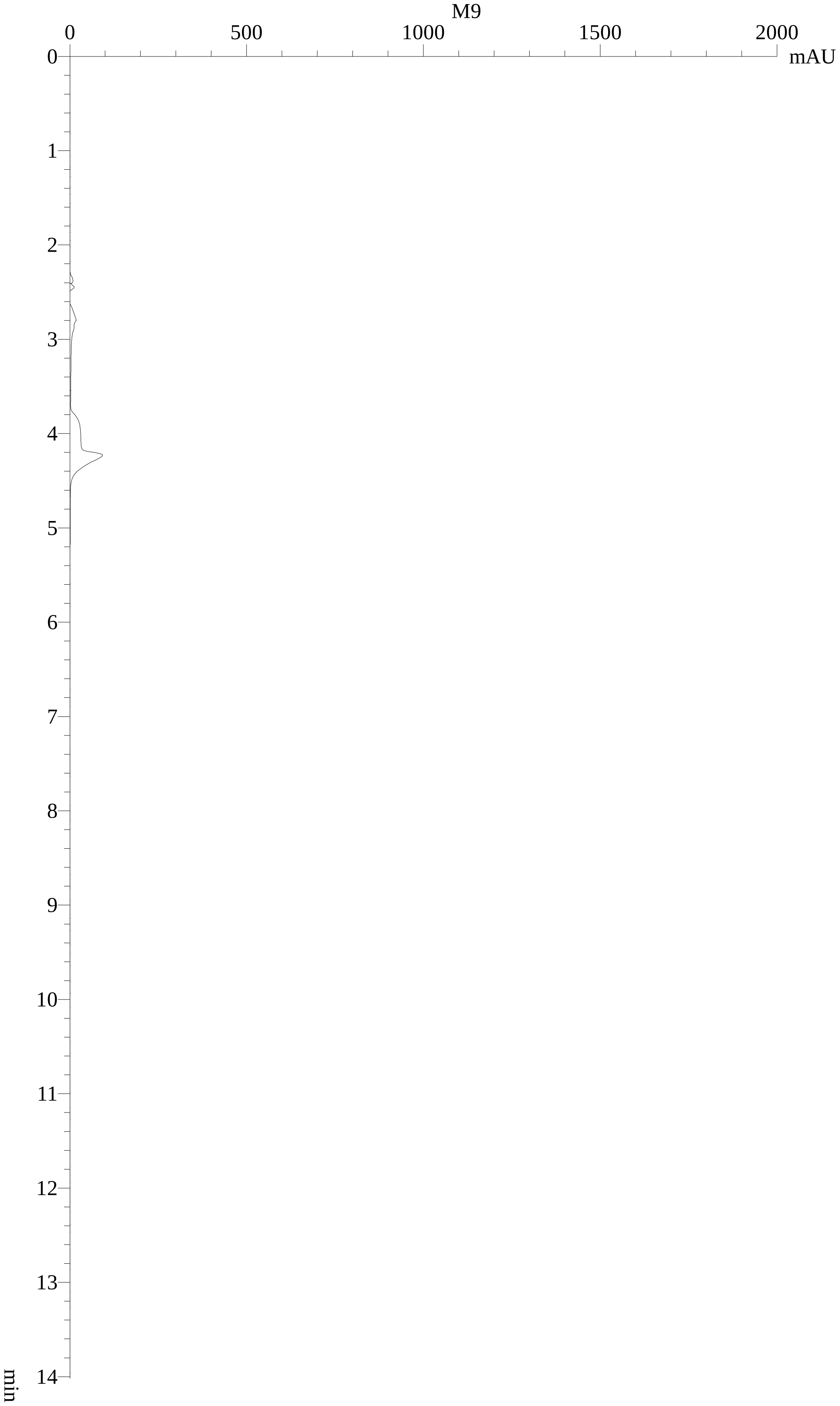
M9
0
500
1000
1500
2000
mAU
0
1
2
3
4
5
6
7
8
9
10
11
12
13
14
min

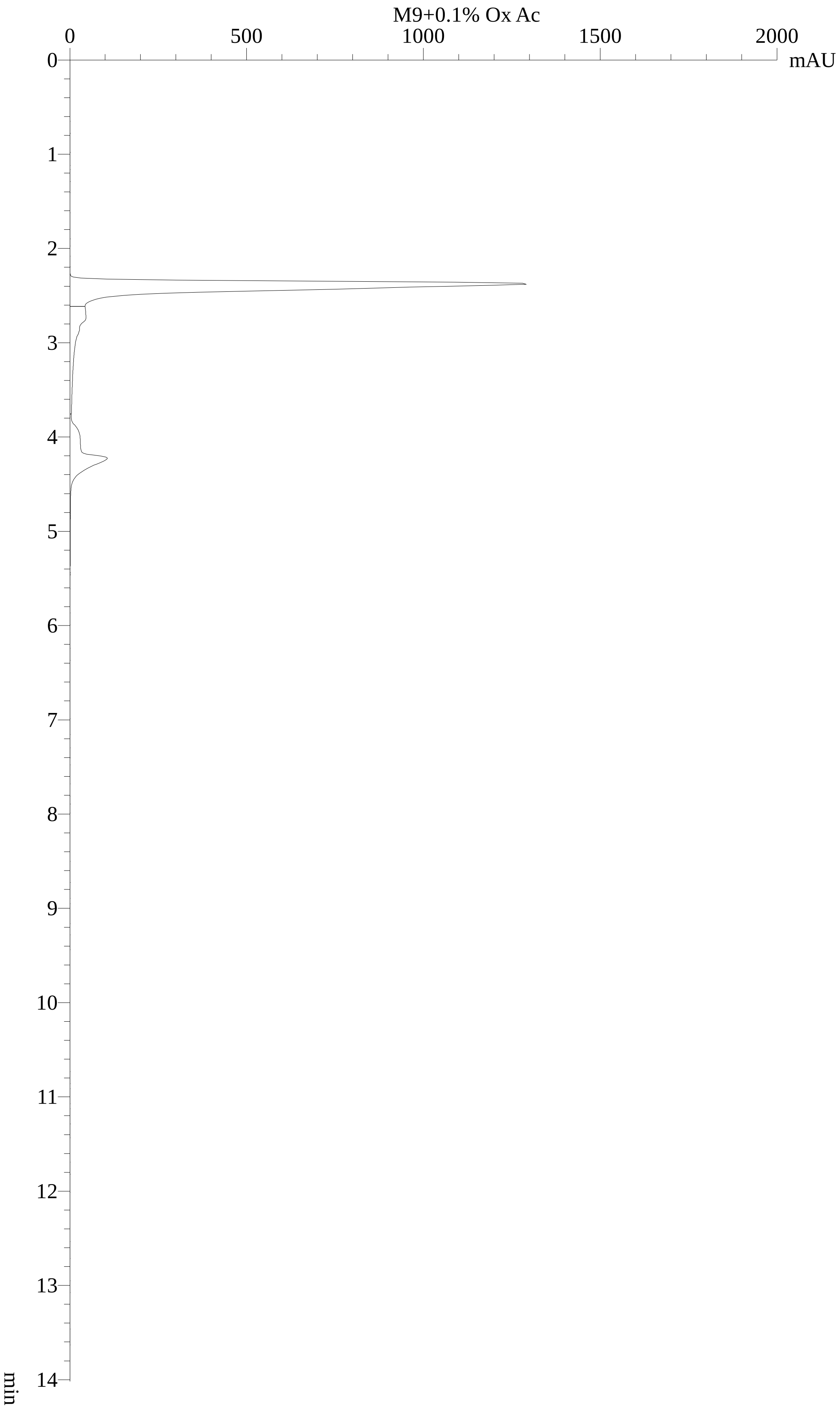
M9+0.1% Ox Ac
0
500
1000
1500
2000
mAU
0
1
2
3
4
5
6
7
8
9
10
11
12
13
14
min

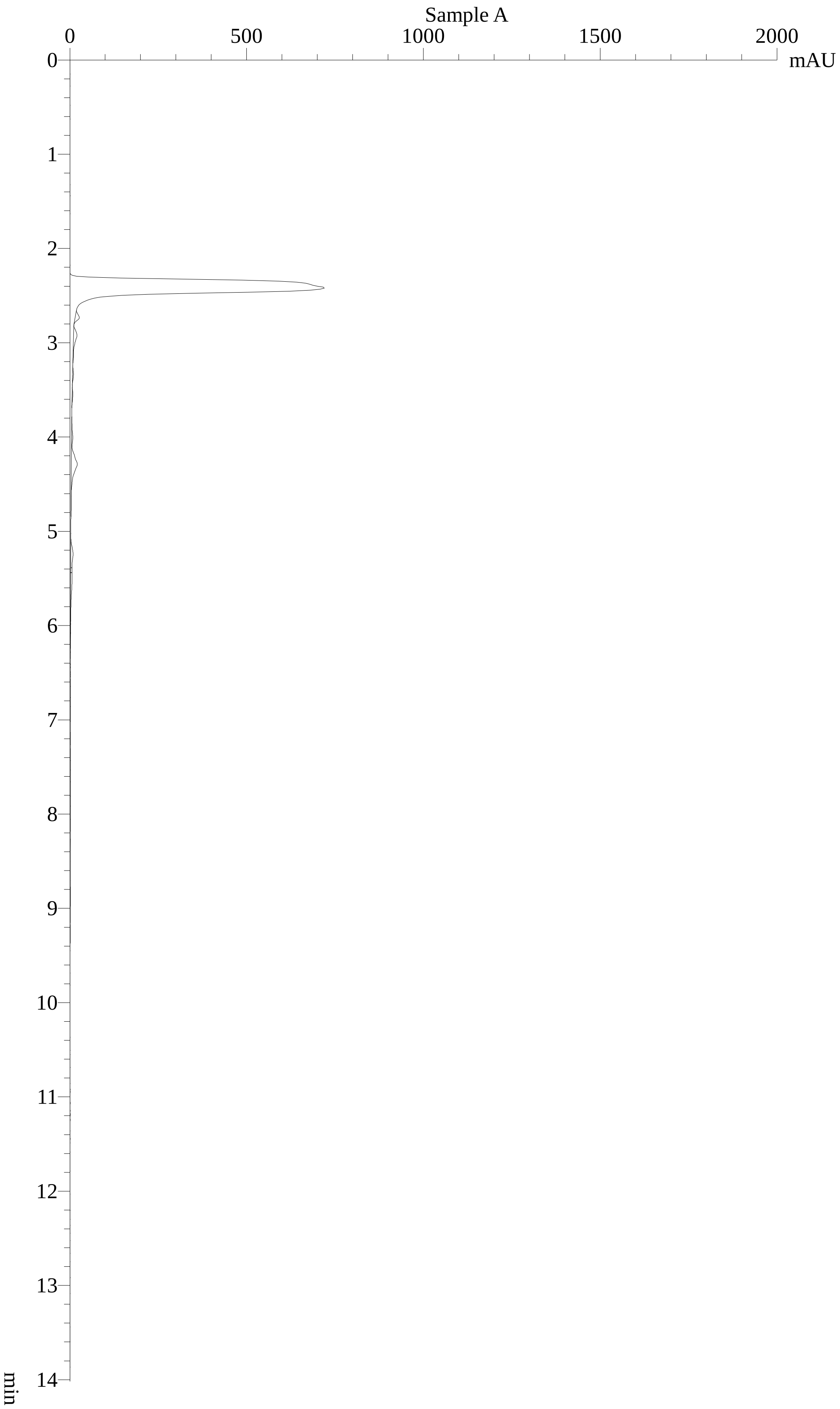
Sample A
0
500
1000
1500
2000
mAU
0
1
2
3
4
5
6
7
8
9
10
11
12
13
14
min

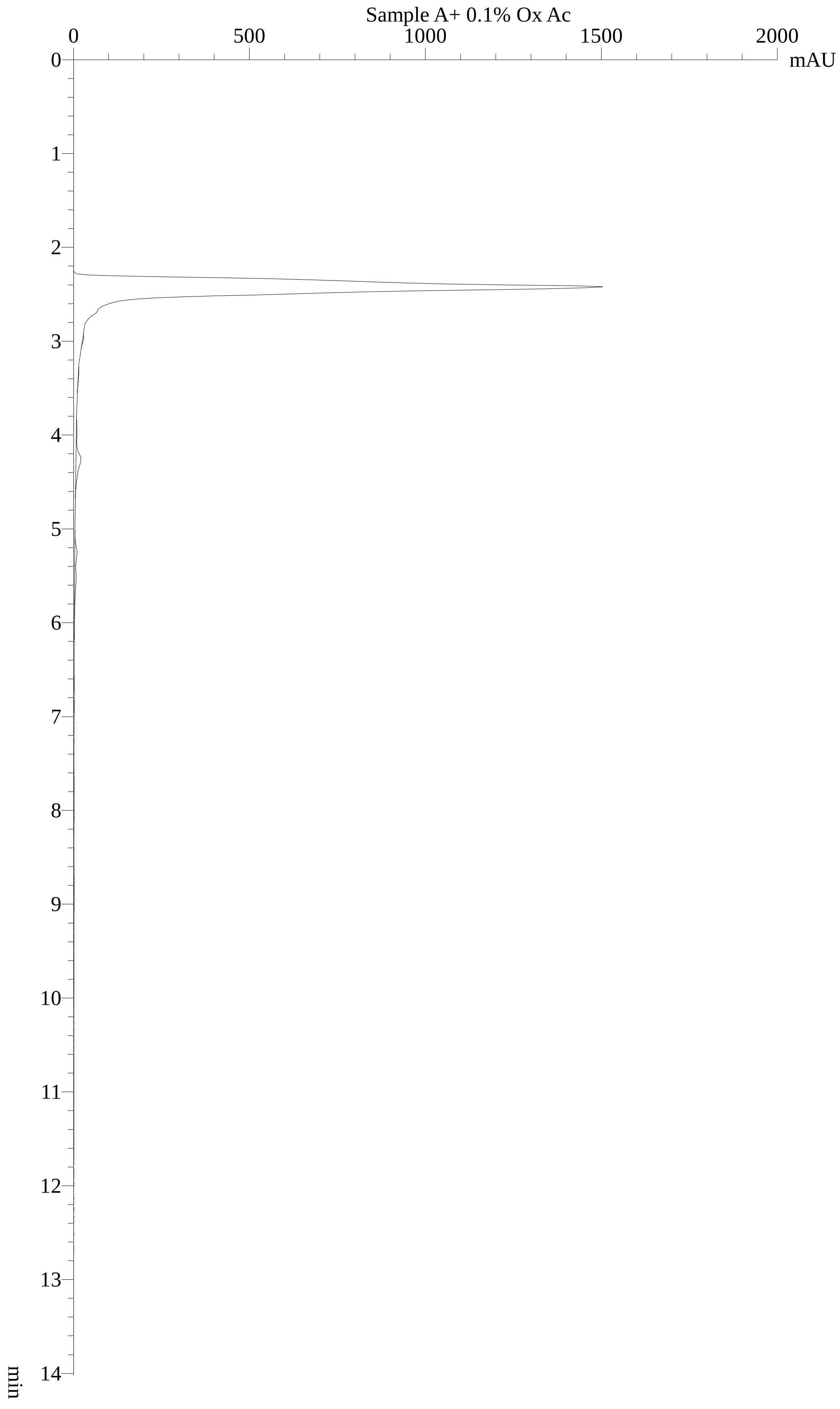

Sample A+ 0.1% Ox Ac
0
500
1000
1500
2000
mAU
0
1
2
3
4
5
6
7
8
9
10
11
12
13
14
min

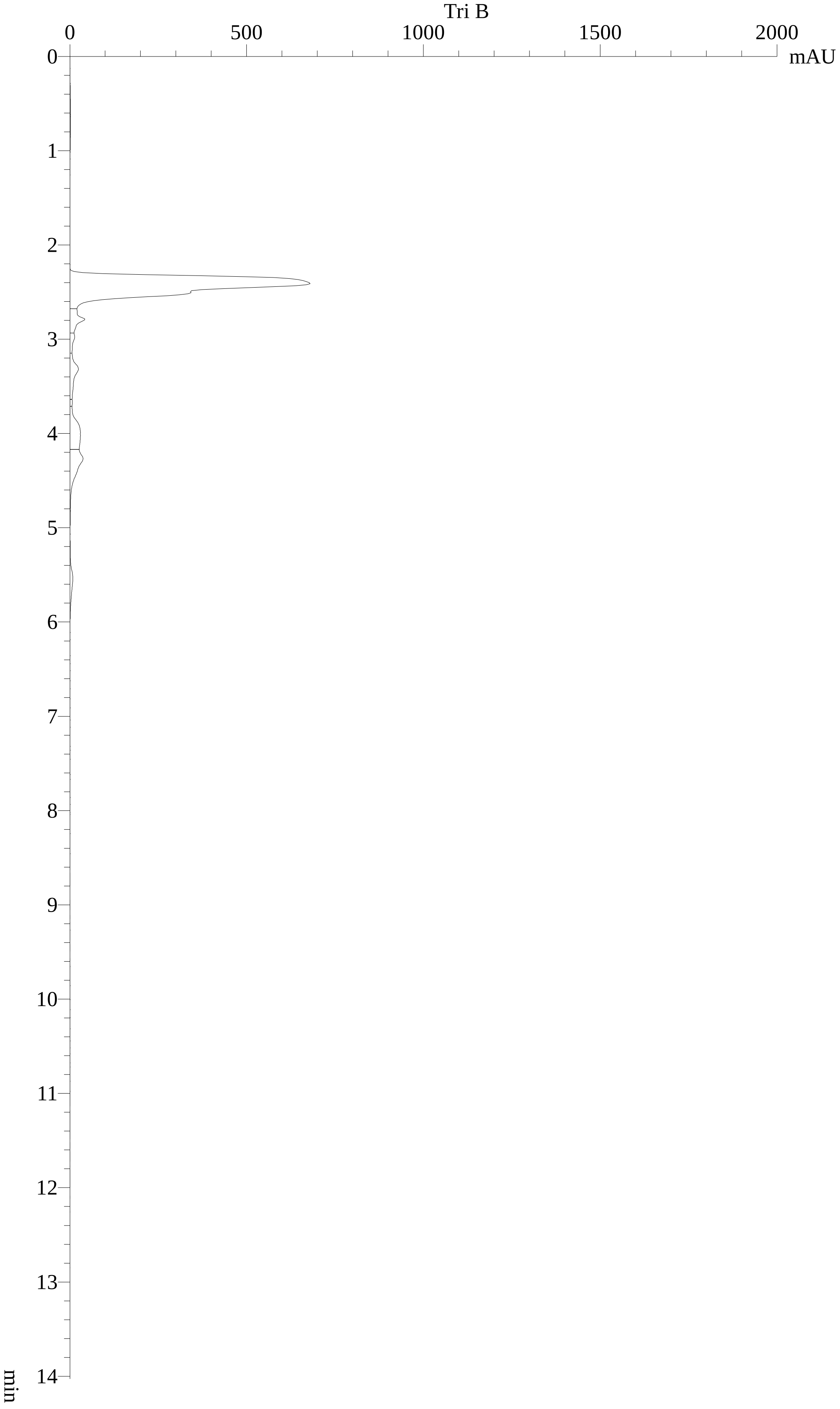
Tri B
0
500
1000
1500
2000
mAU
0
1
2
3
4
5
6
7
8
9
10
11
12
13
14
min

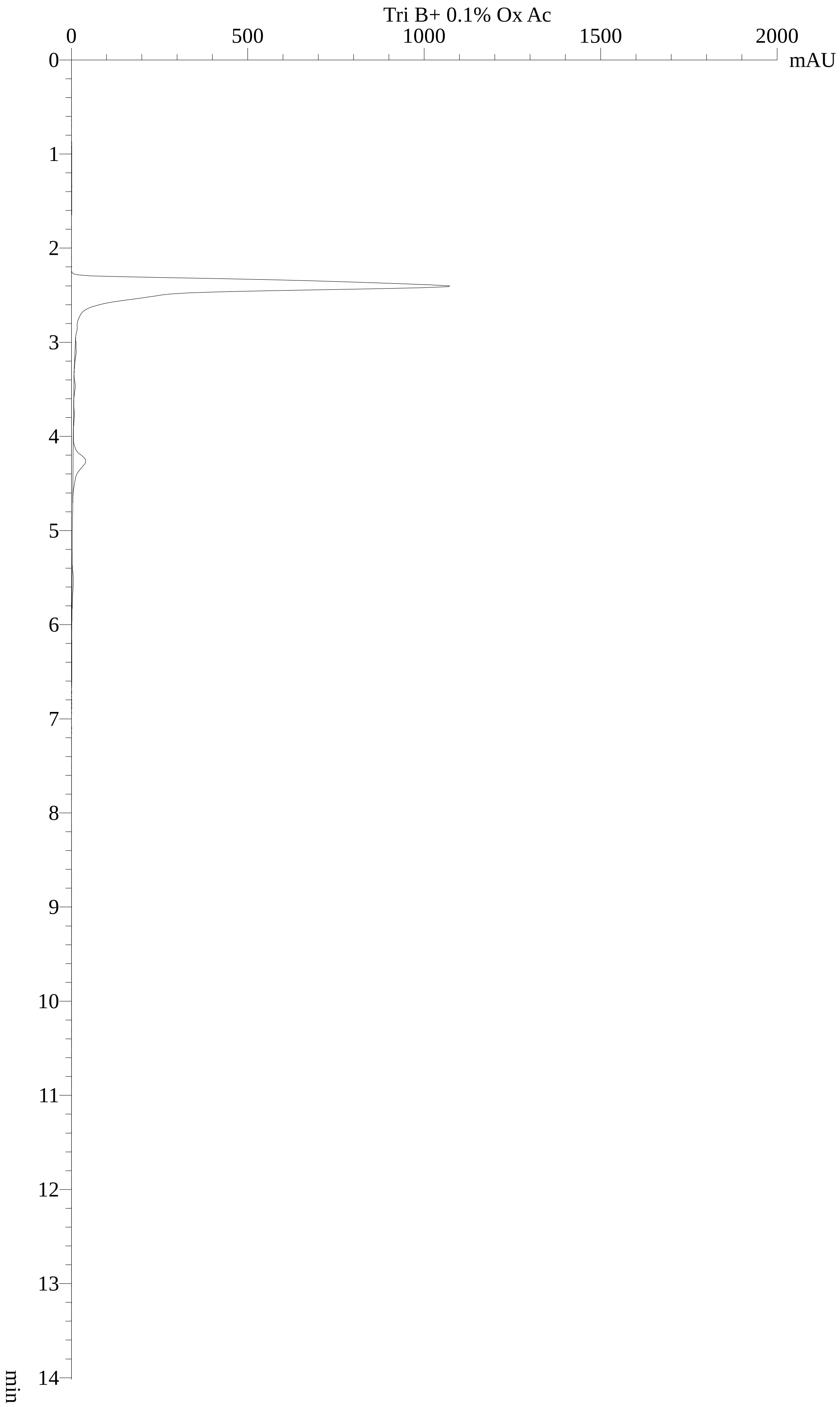
Tri B+ 0.1% Ox Ac
0
500
1000
1500
2000
mAU
0
1
2
3
4
5
6
7
8
9
10
11
12
13
14
min

Oxalic acid cal

ID# : 1
Name : Oxalic acid
Quantitative Method : External Standard
Function : f(x)=8,70380e+007*x+0
Rr1=0,9934920 Rr2=0,9870263 RSS=2,094493e+012
MeanRF: 7,987142e+007 RFSD: 2,688731e+007 RFRSD: 33,663247
FitType : Linear
ZeroThrough : Through
Weighted Regression : None
Detector Name : PDA

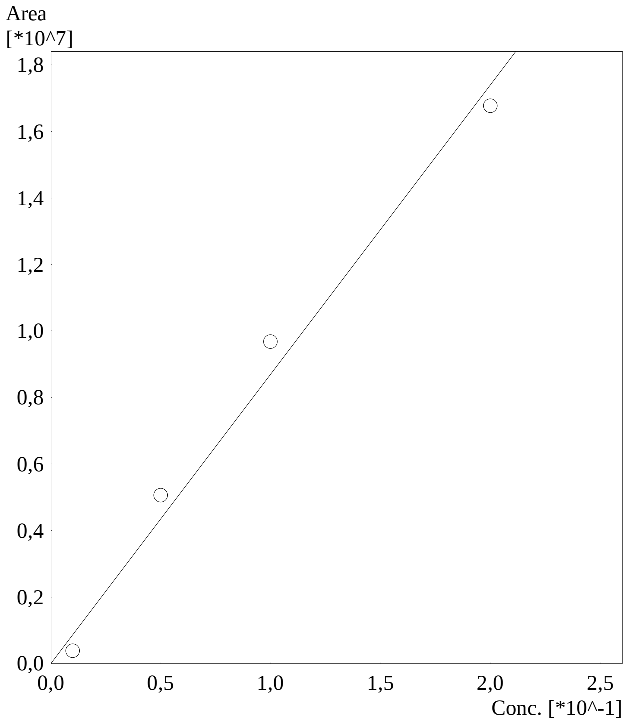

#	Conc.(Ratio)	MeanArea	Area
1	0,01	377914	386101
			369726
2	0,05	5052386	5071290
			5033482
3	0,1	9680204	9400274
			9960133
4	0,2	16768910	16764882
			16772938
