# Supplementary material for: The Response of Paraburkholderia terrae Strains to Two Soil Fungi and the Potential Role of Oxalate
Source: Front Microbiol. 2018 May 29;9:989. doi: 10.3389/fmicb.2018.00989 (PMC5986945; doi:10.3389/fmicb.2018.00989)
Supplement: TABLE S1 — Full factorial ANOVA testing for the effects of bacterial strains, oxalic acid concentration, movement direction, and their interaction on the distance traveled in the chemotaxis experiment on M9-G medium. Statistical significance codes: 0 “∗∗∗” 0.001 “∗∗” 0.01 “∗” 0.05 “.” 0.1 “ ” 1. [file Table_1.DOCX]

**Supplementary Table 1**

**Full factorial ANOVA** testing for the effects of bacterial strains, oxalic acid concentration, movement direction, and their interaction on the distance travelled in the chemotaxis experiment on M9 medium.

**Statistical significance codes: 0 ‘***’ 0.001 ‘**’ 0.01 ‘*’ 0.05 ‘.’ 0.1 ‘ ’ 1**

|  | Df | Sum Sq | Mean Sq | F value | Pr(>F) | Code |
| --- | --- | --- | --- | --- | --- | --- |
| **Direction** | 1 | 1441.0 | 1441.0 | 524.742 | < 2e-16 | *** |
| **Strains (bacterial)** | 5 | 88.0 | 17.6 | 6.409 | 1.78e-05 | *** |
| **Oxalic acid conc.** | 1 | 59.4 | 59.4 | 21.642 | 6.63e-06 | *** |
| **Direction:Strains (bacterial)** | 5 | 18.5 | 3.7 | 1.346 | 0.247477 |  |
| **Direction:Oxalic acid conc.** | 1 | 103.4 | 103.4 | 37.636 | 5.92e-09 | *** |
| **Strain (bacterial) :Oxalic acid conc.** | 5 | 71.3 | 14.3 | 5.190 | 0.000187 | *** |
| **Direction:Strains (bacterial):Oxalic acid conc.** | 5 | 28.4 | 5.7 | 2.065 | 0.072246 | . |
| **Residuals** | 168 | 461.4 | 2.7 |  |  |  |
