# Supplementary material for: The Response of Paraburkholderia terrae Strains to Two Soil Fungi and the Potential Role of Oxalate
Source: Front Microbiol. 2018 May 29;9:989. doi: 10.3389/fmicb.2018.00989 (PMC5986945; doi:10.3389/fmicb.2018.00989)
Supplement: TABLE S2 — Full factorial ANOVA testing for the effects of bacterial strains, fungal strains, exudates level (conc.), movement direction, and their interaction on the distance traveled in the chemotaxis experiment on M9-G medium. Statistical significance codes: 0 “∗∗∗” 0.001 “∗∗” 0.01 “∗” 0.05 “.” 0.1 “ ” 1. [file Table_2.DOCX]

|  | Df | Sum Sq | Mean Sq | F value | Pr(>F) | Code |
| --- | --- | --- | --- | --- | --- | --- |
| **Direction** | 1 | 1211.3 | 1211.3 | 1.085.545 | < 2e-16 | *** |
| **Strains (bacterial)** | 5 | 146.2 | 29.2 | 26.207 | < 2e-16 | *** |
| **Exudates concentration** | 1 | 85.8 | 85.8 | 76.935 | < 2e-16 | *** |
| **Fungal strains** | 1 | 49.6 | 49.6 | 44.446 | 1.07e-10 | *** |
| **Direction: Strain (bacterial)** | 5 | 21.6 | 4.3 | 3.869 | 0.00201 | ** |
| **Direction:Exudates conc.** | 1 | 424.3 | 424.3 | 380.249 | < 2e-16 | *** |
| **Strain (bacterial) :Exudates conc.** | 5 | 10.8 | 2.2 | 1.939 | 0.08738 | . |
| **Direction: Fungal strains** | 1 | 11.3 | 11.3 | 10.166 | 0.00156 | ** |
| **Strain (bacterial): Fungal strains** | 5 | 98.4 | 19.7 | 17.633 | 1.62e-15 | *** |
| **Exudates conc. : Fungal strains** | 1 | 39.8 | 39.8 | 35.631 | 6.06e-09 | *** |
| **Direction:Strain (bacterial):Exudate conc.** | 5 | 16.6 | 3.3 | 2.982 | 0.01195 | * |
| **Direction:Strain (bacterial): Fungal strains** | 5 | 21.6 | 4.3 | 3.876 | 0.00198 | ** |
| **Direction:Exudates conc.: Fungal strains** | 1 | 4.7 | 4.7 | 4.178 | 0.04174 | * |
| **Strain (bacterial):Exudates conc.: Fungal strains** | 5 | 18.1 | 3.6 | 3.249 | 0.00703 | ** |
| **Direction:Strain (bacterial):Exudates conc.:Fungal strains** | 5 | 6.5 | 1.3 | 1.157 | 0.33006 |  |
| **Residuals** | 336 | 374.9 | 1.1 |  |  |  |

**Supplementary Table 2**

**Full factorial ANOVA** testing for the effects of bacterial strains, fungal strains, exudates levels (conc.), movement direction, and their interaction on the distance travelled in the chemotaxis experiment on M9 medium.

**Statistical significance codes: 0 ‘***’ 0.001 ‘**’ 0.01 ‘*’ 0.05 ‘.’ 0.1 ‘ ’ 1**
